# A Case of Systemic Toxicity Related to Intravesical Bacillus Calmette-Guerin for the Treatment of Bladder Cancer

**DOI:** 10.7759/cureus.15321

**Published:** 2021-05-29

**Authors:** Sachin Gupta, Balraj Singh, Harshil Bhatt, Sandeep Singh, Sorab Gupta

**Affiliations:** 1 Hospital Medicine, Tower Health Reading Hospital, West Reading, USA; 2 Hematology/Oncology, Saint Joseph's University Medical Center, Paterson, USA; 3 Internal Medicine, Goshen Hospital, Goshen, USA; 4 Internal Medicine, Indiana University School of Medicine, South Bend, USA; 5 Department of Hematology and Oncology, Bronx Lebanon Hospital, New York, USA

**Keywords:** intravesical bcg, localized bladder cancer, systemic toxicity of bcg, disseminated bcg, immunotherapy in bladder cancer

## Abstract

Intravesical administration of Bacillus Calmette-Guerin (BCG) has been utilized for the treatment of superficial bladder cancer for the past several decades. Though this treatment is well tolerated in general, both local and systematic side effects have been reported. We present a case of a patient who presented with systemic symptoms of fever with chills associated with leucopenia, thrombocytopenia, abnormal liver function tests (LFTs), and splenomegaly a few weeks after an episode of traumatic instillation of intravesical BCG. Though the mycobacterial cultures were negative, he was started on an anti-mycobacterial regimen empirically to which he responded and ultimately fully recovered.

## Introduction

Bacillus Calmette-Guerin (BCG) is the live attenuated strain of bacterium *Mycobacterium bovis* which is generally used as a vaccine against severe tuberculosis infection in the developing world [1]. Over the past several decades, it has been utilized for the treatment of superficial bladder cancer effectively. Induction of strong cell-mediated immune response in the bladder after local intravesical instillation is believed to trigger an attack against the malignant cells and thus providing an effective anti-tumor response [2,3]. Though treatment with intravesical BCG is well-tolerated, both local and systemic side effects have been reported with protean manifestations [4]. Here, we present a case of an elderly patient who presented with signs and symptoms suggestive of disseminated BCG infection shortly after a course of possible traumatic intravesical BCG administration.

## Case presentation

Our patient is a 78-year-old white male with a past medical history of hypertension and benign prostatic hypertrophy (BPH) who had undergone cystoscopy and transurethral resection of bladder tumor by a urologist. The pathology showed non-muscularis propria involving high-grade papillary urothelial carcinoma for which he was undergoing treatment with intravesical BCG administration to prevent a recurrence. He tolerated the initial treatment sessions without any complication but during one of the sessions, he needed multiple attempts to insert the Foley catheter which was painful for him. Subsequently, a “coude” urinary catheter was placed for BCG administration. The patient was hospitalized the same day for intense dysuria and low-grade fevers with chills. At that point, his urinalysis showed elevated white blood cells and red blood cells but no bacteria. He was admitted with a diagnosis of a complicated urinary tract infection (UTI) and initially was started on intravenous Cefepime. He was seen with infectious diseases and was switched to Levofloxacin the next day to cover BCG cystitis and other bacterial organisms for UTI. Cultures remained negative at that time and he was discharged to a rehabilitation facility where he completed 14 days course of Levofloxacin treatment.

The patient complained of intermittent chills and dysuria throughout his stay at the rehabilitation facility where he stayed for almost three weeks. Within three days after discharge from the rehabilitation center, he came back to the emergency department (ED) with severe disabling chills and rigors, fever in the range of 101-103 °F, and generalized weakness. On initial evaluation, he was found to be febrile with a temperature of 101 °F, heart rate of 110 beats per minute, blood pressure of 130/80 mmHg, and pulse oximeter saturation reading of 95% on room air. The patient was very uncomfortable but denied any localizing symptoms like chest pain, shortness of breath, coughing, nausea, vomiting, diarrhea, headache, skin rash, etc. He reported mild dysuria which was an ongoing issue since his last presentation. His blood work showed leucopenia with a white blood cell (WBC) count of 3000/µL (NL 4500-11,000/µL), absolute neutrophil count of 800/µL (NL 2500-7500/µL), thrombocytopenia with a platelet count of 63,000/µL (NL 150,000-450,000/µL), transaminitis with elevated aspartate transaminase (AST) and alanine transaminase (ALT) in the range of 200 unit/liter (NL 5-40 U/L) and elevation in alkaline phosphatase (ALP) 250 U/L (40-150 U/L). A computed tomography (CT) scan of chest, abdomen, and pelvis with contrast was done which showed new moderate splenomegaly. He was started on broad-spectrum antibiotics from ED with intravenous vancomycin, piperacillin/ tazobactam, and levofloxacin. The next day he was examined by various specialists including Infectious diseases, hematology, gastroenterology, and urology. Extensive workup for infectious etiology was ordered including blood and urine cultures, cultures for acid-fast bacilli, Lyme serology, infectious mononucleosis panel, respiratory viral panel, and blood parasite smear, the results for which remained unremarkable. Additional workup by that time involved tagged WBC scan and flow cytometry which were negative as well. Despite being on broad-spectrum antibiotics patient continued to have recurrent fevers in the range of 101-103 °F associated with disabling chills.

There was a strong suspicion of disseminated BCG infection, but the team was hesitant about starting on usual anti-mycobacterial medications because of transaminitis. On day 4, antibiotics were switched to levofloxacin, amikacin, and ethambutol which are all active agents against BCG. The patient’s WBC and platelet counts showed slight improvement and liver function test (LFT) showed some improvement over the course of the next few days and the patient was then transitioned to Ethambutol (dose 15 mg/kg oral per day), Rifampin (dose 600 mg oral per day), Isoniazid (INH; dose 5 mg/kg oral daily)/vitamin B6 after multidisciplinary discussions. The patient showed definitive improvement in symptoms subsequently with improvements in fevers, chills, and rigors and overall started to feel much better. After approximately six days of getting Rifampin, AST/ALT showed an increase to 400 U/L and thus rifampin was stopped and switched to levofloxacin (dose 500 mg oral per day). His dose of INH and Ethambutol was also reduced to one-half of the initial dosage. A decision was made to continue levofloxacin for two months and INH and Ethambutol for six months.

As the patient continued to show symptomatic improvement, he was discharged to a rehabilitation facility after a few days and was followed up as an outpatient with infectious disease and hematology. His cell counts and LFTs eventually normalized and repeat imaging showed complete resolution of splenomegaly.

## Discussion

Cancer of the urinary bladder is the sixth most common malignancy which accounts for 4.6% of all new cancer cases with an estimated around 81,000 new cases in the year 2020 [5]. It is the third most common cancer in men and the eleventh most common cancer in women [6]. Majority of patients with bladder cancer present with non-muscle-invasive bladder cancer, including carcinoma in situ and Ta and T1 stage and thus leading to a good five-year survival rate of around 77% [5]. Morales et al. showed in 1976 that instillation of BCG could be utilized in the treatment of in situ transitional cell carcinoma of the bladder [7]. Since then, local instillation of BCG vaccine has been utilized after the initial transurethral resection of bladder tumor for treatment to prevent a recurrence. The exact mechanism of action of BCG in bladder cancer is not known but it is thought to be related to the attachment of live BCG to the urothelium followed by recruitment of immune cells to the area including granulocytes, macrophages, and lymphocytes, and subsequent release of cytokines which ultimately leads to the death of tumor cells [2,3].

Both local and systemic toxicities have been reported after BCG instillation in these patients. Local complications include cystitis, bladder contracture, bladder ulcerations, balanitis, and prostatitis. Systemic complications though rare have also been reported in case reports and case series. An institutional cohort study published in 2014 showed a cumulative incidence of 4.3% for systemic BCG infection with patients having protean manifestations including military tuberculosis, persistent fever, lymphocytic meningitis, arthritis, and tubule-interstitial nephritis [4]. Also, cases with vascular involvement in the form of mycotic aortic aneurysms [8] as well as BCG-induced small-vessel central nervous system vasculitis has been reported [9] which further point towards different mechanisms of systemic BCG toxicity with direct hematogenous spread of bacteria as one mechanism and inflammatory or immune-mediated mechanism in the other.

Most of the cases of complications related to BCG treatment have been reported within days to few weeks of intra-vesical administration but there are some cases in which delayed presentation with epididymo-orchitis after 17 years [10] and disseminated infection in a post-renal transplant patient after five years of initial treatment with BCG [11] have been reported as well. This points toward the importance of obtaining a detailed history as the very remote history of BCG instillation may get overlooked and the nonspecific presentations and lack of reliable diagnostic means may lead to missing the diagnosis in such cases.

Diagnosis of disseminated BCG is a challenging task due to the rarity of the condition and variable presentation. Also, the fastidious nature of the bacteria makes overall microbiological yield extremely low. In a systematic analysis, the diagnostic performance rates for acid-fast bacilli staining, conventional mycobacterium cultures, and polymerase chain reaction (PCR) based assays were 25.3%, 40.9%, and 41.8%, respectively [4]. The presence of *Mycobacterial bovis* in the liver tissues has been shown with PCR amplification by Leebeek et al. demonstrating direct infection [12]. Others have shown the presence of non-caseating granulomas with eosinophilic infiltration with negative microbiological evidence pointing toward hypersensitivity reaction as etiology [13].

Similarly, there are no specific guidelines for the management of disseminated BCG but most of the cases were treated with a combination of anti-tubercular regimen plus steroids empirically. Different routes and dosages of steroids have been used and for the variable duration in different case reports/series. Similarly, there is no consensus regarding the choice for an anti-tubercular regimen. At the same time, the presence of multiple organ dysfunction including derangement of LFTs makes the initiation of standard anti-tubercular regimen challenging. In our patient, we were not able to start the standard anti-tubercular regimen until some improvement in LFTs was noted and still we had to interrupt the use of INH at one point as LFTs trended upward after a few days of initiation. Contrary to other care reports, we did not use prednisone or other steroids in our patient as we were strongly suspecting disseminated BCG rather than hypersensitivity reaction since his problems started immediately after the episode of traumatic catheterization for BCG instillation. Moreover, we were cautious regarding immune suppression as the patient was already neutropenic. It is still debatable if the use of steroids in this situation would have fastened the recovery. Our patient showed overall improvement in his symptoms after initiation of an anti-tubercular regimen and was followed very closely for regular blood work which showed continued improvement.

There have been studies to reduce the toxicity associated with BCG instillation. A recent study with a lower dose of BCG and a lesser duration of therapy has shown no significant differences in the side effects [14]. Similarly, a study that utilized prophylactic administration of Isoniazid did not show any significant improvement in the incidence of cystitis, frequency, hematuria, or severe fever [15]. Whereas another study utilizing ofloxacin showed some improvement in the incidence of moderate and severe adverse events associated with BCG usage [16]. As in our patient, traumatic catheterization during BCG instillation appears to be an important risk factor and proper precautions at that time may lead to a reduction in the incidence of adverse events associated with BCG therapy.

## Conclusions

Intravesical instillation of BCG for non-muscle invasive bladder cancer has proved to be highly effective and is being utilized for over four decades. Both local and systemic complications have been reported. The exact pathological mechanism leading to systemic sepsis-like presentation is not clear and further information is needed for this. At the same time, no definitive treatment guidelines are available. This further makes the therapeutic decisions regarding the initiation of appropriate anti-tubercular regimens challenging especially in patients who present with multi-system involvement given the side-effect profile of the anti-tubercular regimen. The use of steroids is again questionable, as our patient improved without the use of steroids. Being aware of the current and past therapy with intravesical BCG and the complications associated with it helps in the early identification of the syndrome. Early mobilization of a multi-disciplinary team including infectious diseases, hematology, oncology, and gastroenterology is needed and was helpful in our patient.
